# Supplementary material for: An Insight into the Exploration of Antibiotic Resistance Genes in Calorie Restricted Diet Fed Mice
Source: Nutrients. 2023 Jul 19;15(14):3198. doi: 10.3390/nu15143198 (PMC10384538; doi:10.3390/nu15143198)
Supplement: Supplementary file 1 [file nutrients-15-03198-s001.zip › nutrients-2480204-supplementary.pdf]

Supplementary Material

An insight into the exploration of antibiotics resistance genes in calorie restricted diet fed mice

Xiuqin Fan [1], Yuanyuan Lu [2], Yunfeng Zhao [3], Hongjian Miao [3,*], Kemin Qi [1] and Rui Wang [1,*]

1 Laboratory of Nutrition and Development, Key Laboratory of Major Diseases in Children, Ministry of Education, Beijing Pediatric Research Institute, Beijing Children's Hospital, Capital Medical University, National Center for Children's Health, Beijing 100045, China; qincaifan@126.com (X.F.); qikemin@bch.com.cn (K.Q.)

2 Department of Children's Health Care Center, Beijing Children'sHospital, Capital Medical University, National Center for Children's Health, Beijing 100045, China; luyuan1988220@163.com

3 NHC Key Laboratory of Food Safety Risk Assessment, Chinese Academy of Medical Sciences Research Unit (2019RU014), China National Center for Food Safety Risk Assessment, Beijing 100021, China; zhaoyf@cfsa.net.cn

* Correspondence: miaohj@cfsa.net.cn (H.M.); 15810948239@163.com (R.W.);

Tel.: +86-10-67779768 (H.M.); +86-10-59616895 (R.W.)

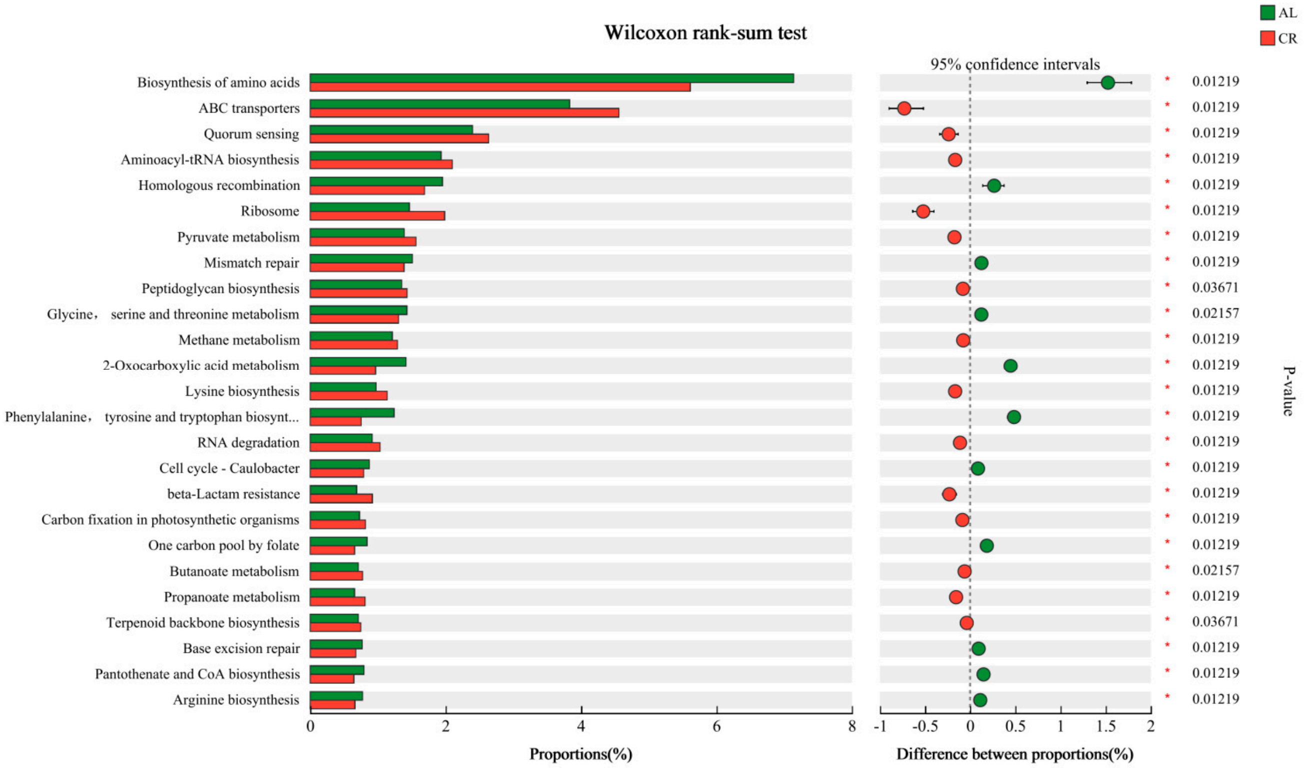

Fig. S1 Wilcoxon rank-sum test of functional genes in mouse feces between AL and CR groups.

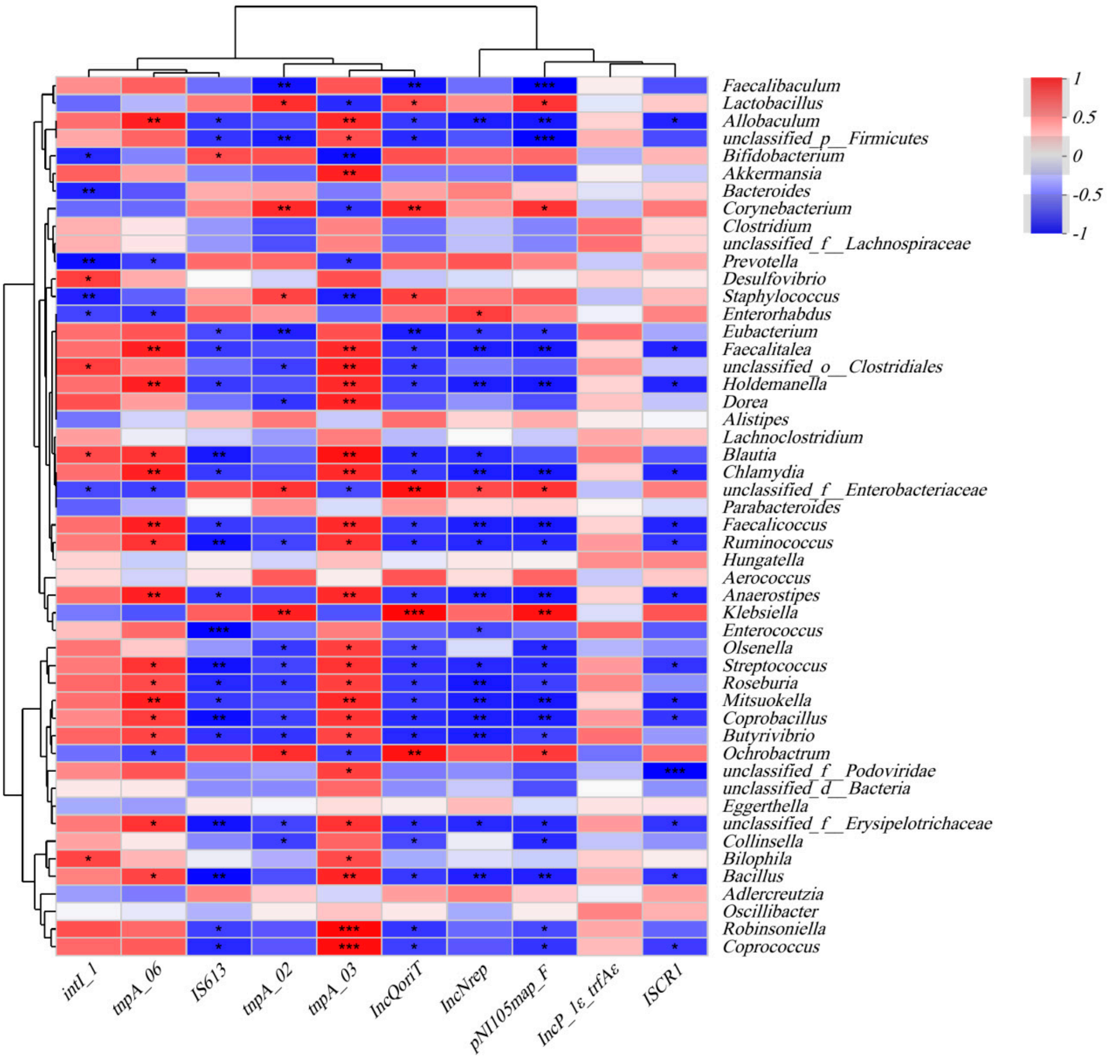

Fig. S2 The Spearman correlations between gut microbiota and MGEs (at Genus level).

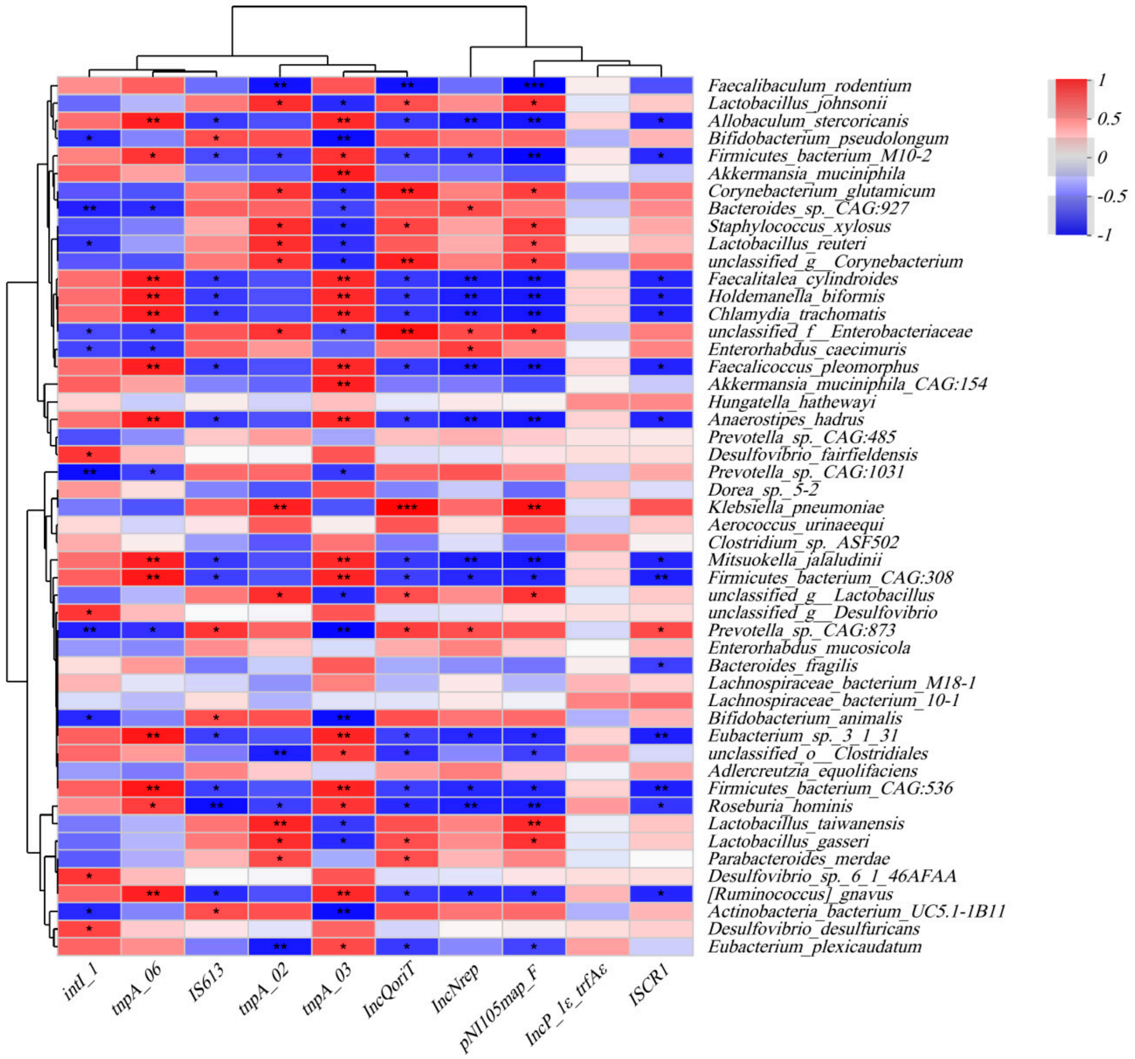

Fig. S3 The Spearman correlations between gut microbiota and MGEs (at Species level).

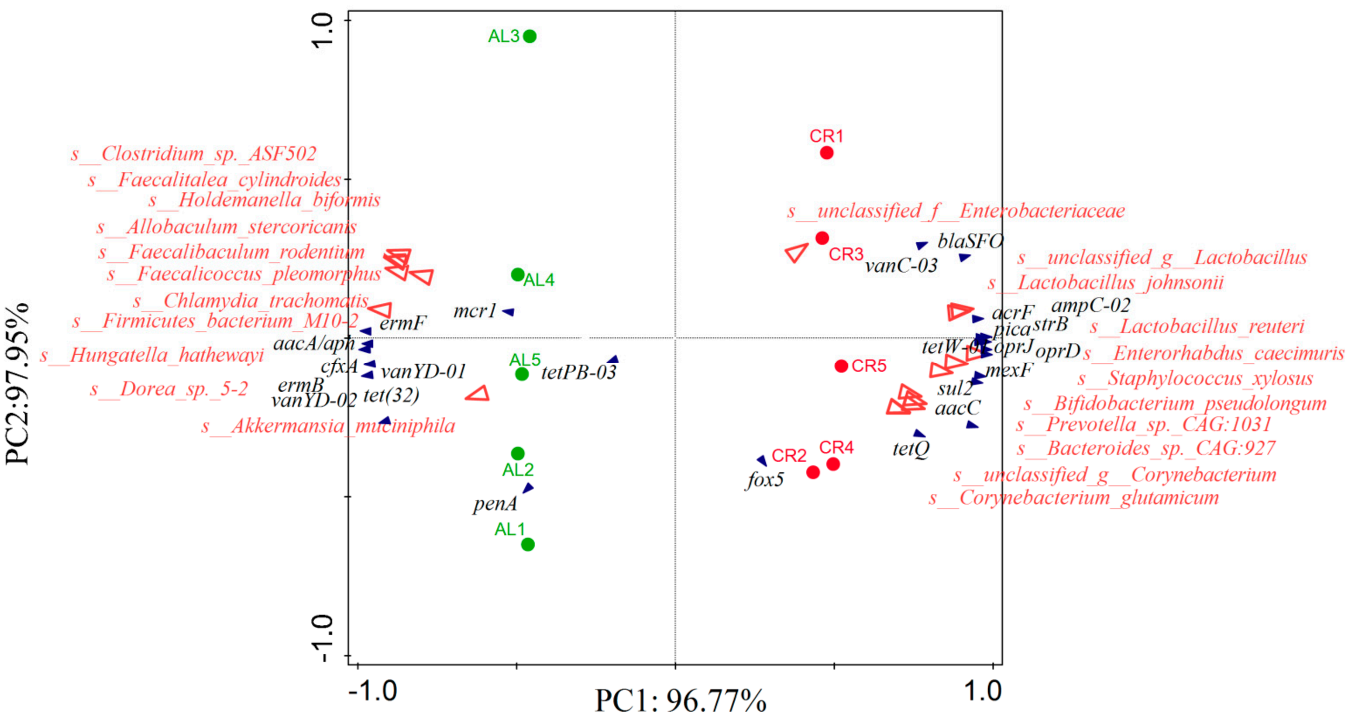

Fig. S4 RDA analysis between ARGs and gut microbiota in mouse feces (at Speceis level).

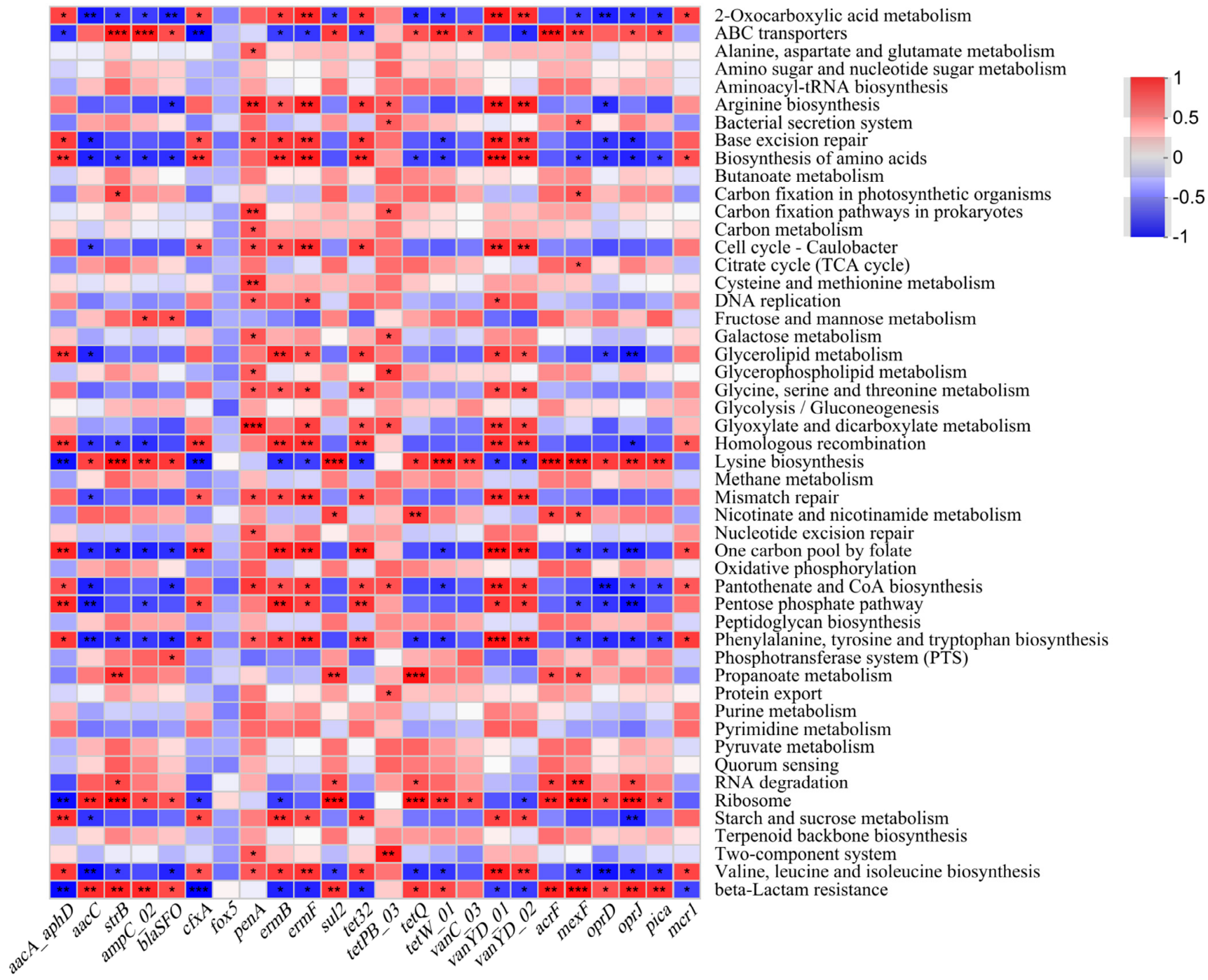

Fig. S5 The Spearman correlations between ARGs and abundance of functional genes (at 3 level).

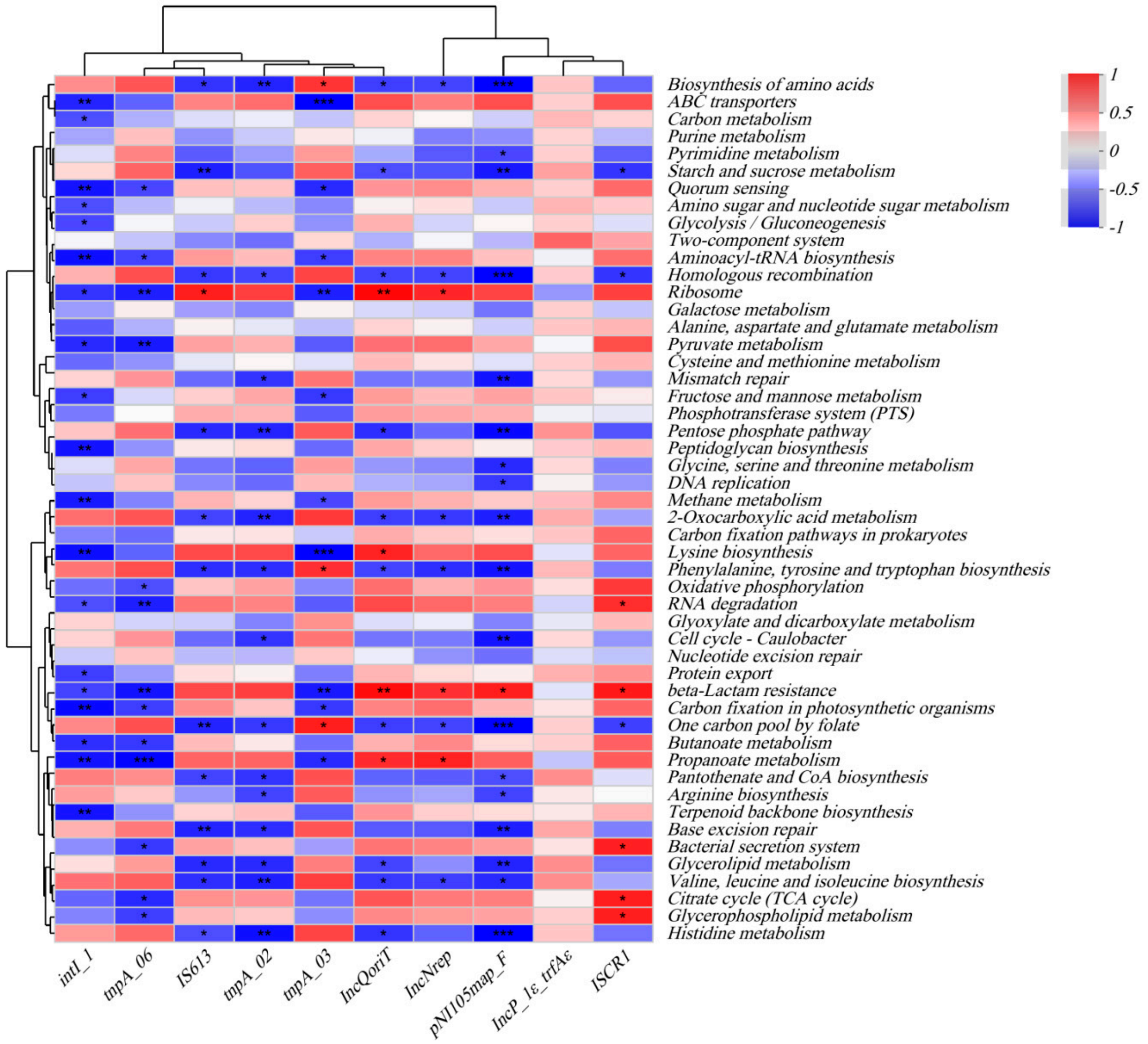

Fig. S6 The Spearman correlations between MGEs and abundance of functional genes (at 3 level).

Table S1 Details of 88 pairs of primers for high-throughput quantitative PCR (HT-qPCR) [1].

Assay Name	Gene Name	Mechanisms	Types	Forward	Reverse
16S rRNA	16S rRNA			GGGTTGCGCTCGTTGC	ATGGYTGTCGTCAGCTCGTG
aac	aac	antibiotic inactivation	Aminoglycoside	CCCTGCGTTGTGGCTATGT	TTGGCCACGCCAATCC
aacA/aphD	aacA/aphD	antibiotic inactivation	Aminoglycoside	AGAGCCTTGGGAAGATGAAGTTT	TTGATCCATACCATAGACTATCTCATCA
aacC	aacC	antibiotic inactivation	Aminoglycoside	CGTCACTTATTCGATGCCCTTAC	GTCGGGCGCGGCATA
aadA-02	aadA	antibiotic inactivation	Aminoglycoside	CGAGATTCTCCGCGCTGTA	GCTGCCATTCTCCAAATTGC
aphA3-02	aphA3	antibiotic inactivation	Aminoglycoside	CGGAATTGAAAAAACTGATCGAA	ATACCGGCTGTCCGTCATTT
aadA2-03	aadA2	antibiotic inactivation	Aminoglycoside	CAATGACATTCTTGCGGGTATC	GACCTACCAAGGCAACGCTATG
aadA5-02	aadA5	antibiotic inactivation	Aminoglycoside	GTTCTTGCTCTTGCTCGCATT	GATGCTCGGCAGGCAAAC
aadA9-01	aadA9	antibiotic inactivation	Aminoglycoside	CGCGGCAAGCCTATCTTG	CAAATCAGCGACCGCAGACT
aadE	aadE	antibiotic inactivation	Aminoglycoside	TACCTTATTGCCCTTGGAAGAGTTA	GGAACTATGTCCCTTTTAATTCTACAATCT
aph(2')-Id-01	aph(2')-Id	antibiotic inactivation	Aminoglycoside	TGAGCAGTATCATAAGTTGAGTGAAAAG	GACAGAACAATCAATCTCTATGGAATG
strB	strB	antibiotic inactivation	Aminoglycoside	GCTCGGTCGTGAGAACAATCT	CAATTTCGGTCGCCTGGTAGT
ampC-01	ampC	antibiotic inactivation	Beta-Lactamase	TGGCGTATCGGGTCAATGT	CTCCACGGGCCAGTTGAG
ampC-02	ampC	antibiotic inactivation	Beta-Lactamase	GCAGCACGCCCCGTAA	TGTACCCATGATGCGCGTACT
ampC-04	ampC	antibiotic inactivation	Beta-Lactamase	TCCGGTGACGCGACAGA	CAGCACGCCGGTGAAAGT
blaCMY2-02	blaCMY2	antibiotic inactivation	Beta-Lactamase	GCGAGCAGCCTGAAGCA	CGGATGGGCTTGTCCTCTT
blaCTX-M-04	blaCTX-M	antibiotic inactivation	Beta-Lactamase	CTTGGCGTTGCGCTGAT	CGTTCATCGGCACGGTAGA
blaOXY	blaOXY	antibiotic inactivation	Beta-Lactamase	CGTTCAGGCGGCAGGTT	GCCGCGATATAAGATTTGAGAATT
blaSFO	blaSFO	antibiotic inactivation	Beta-Lactamase	CCGCCGCCATCCAGTA	GGGCCGCCAAGATGCT
blaSHV-01	blaSHV	antibiotic inactivation	Beta-Lactamase	TCCCATGATGAGCACCTTTAAA	TTCGTCACCGGCATCCA
cfxA	cfxA	antibiotic inactivation	Beta-Lactamase	TCATTCCTCGTTCAAGTTTTCAGA	TGCAGCACCAAGAGGAGATGT
cphA-01	cphA	antibiotic inactivation	Beta-Lactamase	GCGAGCTGCACAAGCTGAT	CGGCCCAGTCGCTCTTC
cphA-02	cphA	antibiotic inactivation	Beta-Lactamase	GTGCTGATGGCGAGTTTCTG	GGTGTGGTAGTTGGTGTTGATCAC
fox5	fox5	antibiotic inactivation	Beta-Lactamase	GGTTTGCCGCTGCAGTTC	GCGGCCAGGTGACCAA
penA	penA	antibiotic target replacement	Beta-Lactamase	AGACGGTAACGTATAACTTTTTGAAAGA	GCGTGTAGCCGGCAATG
blaNDM	blaNDM	antibiotic inactivation	Beta-Lactamase	GGCCACACCAGTGACAATATCA	CAGGCAGCCACCAAAAGC
ermB	ermB	antibiotic target alteration	MLSB	TAAAGGGCATTTAACGACGAAACT	TTTATACCTCTGTTTGTTAGGGAATTGAA
ermF	ermF	antibiotic target alteration	MLSB	CAGCTTTGGTTGAACATTTACGAA	AAATTCCTAAAATCACAACCGACAA
lnuA-01	lnuA	antibiotic inactivation	MLSB	TGACGCTCAACACACTCAAAAA	TTCATGCTTAAGTTCCATACGTGAA

lnuC	lnuC	antibiotic inactivation	MLSB	TGGTCAATATAACAGATGTAAACCAGATTT	CACCCCAGCCACCATCAA
matA/mel	matA/mel	antibiotic efflux	MLSB	TAGTAGGCAAGCTCGGTGTTGA	CCTGTGCTATTTTAAGCCTTGTTTCT
mefA	mefA	antibiotic efflux	MLSB	CCGTAGCATTGGAACAGCTTTT	AAACGGAGTATAAGAGTGCTGCAA
mphA-01	mphA	antibiotic inactivation	MLSB	CTGACGCGCTCCGTGTT	GGTGGTGCATGGCGATCT
oleC	oleC	antibiotic efflux	MLSB	CCCGGAGTCGATGTTCGA	GCCGAAGACGTACACGAACAG
vgaB-01	vgaB	antibiotic target protection	MLSB	TAAAAGAGAATAAGGCGCAAGGA	TGTTTAGTAGCATGTTGCATTTTCC
vgb-01	vgb	antibiotic inactivation	MLSB	AGGGAGGGTATCCATGCAGAT	ACCAAATGCGCCCGTTT
tet(32)	tet(32)	antibiotic target protection	Tetracycline	CCATTACTTCGGACAACGGTAGA	CAATCTCTGTGAGGGCATTTAACA
tet(34)	tet(34)	antibiotic inactivation	Tetracycline	CTTAGCGCAAACAGCAATCAGT	CGGTGATACAGCGCGTAAACT
tetA-02	tetA	antibiotic efflux	Tetracycline	CTCACCAGCCTGACCTCGAT	CACGTTGTTATAGAAGCCGCATAG
tetG-01	tetG	antibiotic efflux	Tetracycline	TCAACCATTGCCGATTCGA	TGGCCCGGCAATCATG
tetG-02	tetG	antibiotic efflux	Tetracycline	CATCAGCGCCGGTCTTATG	CCCCATGTAGCCGAACCA
tetM-01	tetM	antibiotic target protection	Tetracycline	CATCATAGACACGCCAGGACATAT	CGCCATCTTTTGCAGAAATCA
tetM-02	tetM	antibiotic target protection	Tetracycline	TAATATTGGAGTTTTAGCTCATGTTGATG	CCTCTCTGACGTTCTAAAAGCGTATTAT
tetPA	tetPA	antibiotic efflux	Tetracycline	AGTTGCAGATGTGTATAGTCGTAAACTATCTATT	TGCTACAAGTACGAAAACAAAACTAGAA
tetPB-03	tetPB	antibiotic target protection	Tetracycline	TGGGCGACAGTAGGCTTAGAA	TGACCCTACTGAAACATTAGAAATATACCT
tetQ	tetQ	antibiotic target protection	Tetracycline	CGCCTCAGAAGTAAGTTCATACACTAAG	TCGTTCATGCGGATATTATCAGAAT
tetS	tetS	antibiotic target protection	Tetracycline	TTAAGGACAAACTTTCTGACGACATC	TGTCTCCCATTGTTCTGGTTCA
tetW-01	tetW	antibiotic target protection	Tetracycline	ATGAACATTCCCACCGTTATCTTT	ATATCGGCGGAGAGCTTATCC
vanB-01	vanB	antibiotic target alteration	Vancomycin	TTGTCGGCGAAGTGGATCA	AGCCTTTTTCCGGCTCGTT
vanC-03	vanC	antibiotic target alteration	Vancomycin	AAATCAATACTATGCCGGGCTTT	CCGACCGCTGCCATCA
vanC2/vanC3	vanC2/vanC3	antibiotic target alteration	Vancomycin	TTTGACTGTCGGTGCTTGTGA	TCAATCGTTTCAGGCAATGG
vanHB	vanHB	antibiotic target alteration	Vancomycin	GAGGTTTCCGAGGCGACAA	CTCTCGGCGGCAGTCGTAT
vanTC-02	vanTC	antibiotic target alteration	Vancomycin	ACAGTTGCCGCTGGTGAAG	CGTGGCTGGTCGATCAAAA
vanYD-01	vanYD	antibiotic target alteration	Vancomycin	AAGGCGATACCCTGACTGTCA	ATTGCCGGACGGAAGCA
vanYD-02	vanYD	antibiotic target alteration	Vancomycin	CAAACGGAAGAGAGGTCACTTACA	CGGACGGTAATAGGGACTGTTC
acrA-03	acrA	antibiotic efflux	Multidrug	CAGACCCGCATCGCATATT	CGACAATTTCGCGCTCATG
acrA-04	acrA	antibiotic efflux	Multidrug	TACTTTGCGCGCCATCTTC	CGTGCGCGAACGAACAT
acrA-05	acrA	antibiotic efflux	Multidrug	CGTGCGCGAACGAACA	ACTTTGCGCGCCATCTTC

acrF	acrF	antibiotic efflux	Multidrug	GCGGCCAGGCACAAAA	TACGCTCTTCCCACGGTTTC
ceoA	ceoA	antibiotic efflux	Multidrug	ATCAACACGGACCAGGACAAG	GGAAAGTCCGCTCACGATGA
emrD	emrD	antibiotic efflux	Multidrug	CTCAGCAGTATGGTGGTAAGCATT	ACCAGGCGCCGAAGAAC
floR	floR	antibiotic efflux	Multidrug	ATTGTCTTCACGGTGTCCGTTA	CCGCGATGTCGTCGAACT
mepA	mepA	antibiotic efflux	Multidrug	ATCGGTCGCTCTTCGTTCAC	ATAAATAGGATCGAGCTGCTGGAT
mexF	mexF	antibiotic efflux	Multidrug	CCGCGAGAAGGCCAAGA	TTGAGTTCGGCGGTGATGA
mtrD-03	mtrD	antibiotic efflux	Multidrug	CCGCCAAGCCGATATAGACA	GGCCGGGTTGCCAAA
oprD	oprD	antibiotic efflux	Multidrug	ATGAAGTGGAGCGCCATTG	GGCCACGGCGAACTGA
oprJ	oprJ	antibiotic efflux	Multidrug	ACGAGAGTGGCGTCGACAA	AAGGCGATCTCGTTGAGGAA
qacEdelta1-02	qacEΔ1	antibiotic efflux	Multidrug	CCCCTTCCGCCGTTGT	CGACCAGACTGCATAAGCAACA
ttgB	ttgB	antibiotic efflux	Multidrug	TCGCCCTGGATGTACACCTT	ACCATTGCCGACATCAACAAC
yceL/mdtH-01	yceL/mdtH	antibiotic efflux	Multidrug	TCGGGATGGTGGGCAAT	CGATAACCGAGCCGATGTAGA
intI-1	intI-1	MGEs/integrase	MGEs	CGAACGAGTGGCGGAGGGTG	TACCCGAGAGCTTGGCACCCA
tnpA-06	tnpA	MGEs/Transposase- IS6	MGEs	TGCAGATGGTTTAACCTTGGATATTT	TCGGTTCATCAAACTGCTTCAC
IS613	IS613	MGEs/Transposase- IS613	MGEs	AGGTTCGGACTCAATGCAACA	TTCAGCACATACCGCCTTGAT
tnpA-02	tnpA	MGEs/Transposase- IS4 Group	MGEs	GGGCGGGTCGATTGAAA	GTGGGCGGGATCTGCTT
tnpA-03	tnpA	MGEs/Transposase- IS6 Group	MGEs	AATTGATGCGGACGGCTTAA	TCACCAAACTGTTTATGGAGTCGTT
IncQoriT	IncQoriT	MGEs/plasmids	MGEs	TTCGCGCTCGTTGTTCTTCGAGC	GCCGTTAGGCCAGTTTCTCG
IncNrep	IncNrep	MGEs/plasmids	MGEs	AGTTCACCACCTACTCGCTCCG	CAAGTTCTTCTGTTGGGATTCCG
pNI105map-F	pNI105map-F	MGEs/plasmids	MGEs	CCCCCAGGACTTGCGAGCG	GAGGCATGCACGCCGACCA
IncP-1ε-trfAε	IncP-1ε	MGEs/plasmids	MGEs	ACGAAGAAATGGTTGTCCTGTTC	CGTCAGCTTGCGGTACTTCTC
ISCR1	ISCR1	MGEs	MGEs	ATGGTTTCATGCGGGTT	CTGAGGGTGTGAGCGAG
sul2	sul2	antibiotic target replacement	Sulfonamide	TCATCTGCCAAACTCGTCGTTA	GTCAAAGAACGCCGCAATGT
nisB	nisB	unknown	Others	GGGAGAGTTGCCGATGTTGTA	AGCCACTCGTTAAAGGGCAAT
pica	pica	other/unknown	Others	GCAATCGAGGCGGTGTTC	TTGCCGCAGCCAATTCA
sat4	sat4	antibiotic inactivation	Others	GAATGGGCAAAGCATAAAAACTTG	CCGATTTTGAAACCACAATTATGATA
mcr1	mcr1	antibiotic target alteration	Others	CACATCGACGGCGTATTCTG	CAACGAGCATACCGACATCG
22S rDNA	22S rDNA	pathogens/Enterococci	Enterococci	AGAAATTCCAAACGAACTTG	CAGTGCTCTACCTCCATCATT
ecfX	ecfX	pathogens/P. aeruginosa	P. aeruginosa	AGCGTTCGTCCTGCACAAGT	TCCACCATGCTCAGGGAGAT

uidA	uidA	pathogens/E. coli	E. coli	AACCACGCGTCTGTTGACTG	CCCGGTTGCCAGAGGTG

[1] Wang R, Chen D, Wang F, Fan X, Fan C, Tang T, et al. An insight into the exploration of proliferation of antibiotic resistance genes in high-fat diet induced obesity mice. Genomics. 2021;113:2503-12.
